# Commensal *Peptoniphilus harei* induce activation of monocytes via TLR2/CD14 signalling in whole blood

**DOI:** 10.1007/s00430-025-00859-7

**Published:** 2025-11-27

**Authors:** Tobias Schmidt, Inga-Maria Frick, Lotta Happonen, Ariane Neumann.

**Affiliations:** 1 Division of Pediatrics, Clinical Sciences, Lund University, Lund, Sweden; 2 Division of Infection Medicine, Clinical Sciences, Lund University, Lund, Sweden; 3 Wallenberg Center for Molecular Medicine, Lund University, Lund, Sweden

**Keywords:** Commensals, Monocyte activation, Anaerobic cocci, *Peptoniphilus* spp.

## Abstract

Myeloid cells, such as monocytes, are central in the immune response to infections. However, limited knowledge exists regarding the inflammatory response to Gram-positive anaerobic cocci (GPAC).

To explore the ability of GPACs to induce monocyte activation, we compared two GPAC species, *Parvimonas micra* and *Peptoniphilus harei*, with *E. coli,* a major cause of bloodstream infections. We stimulated whole blood with heat-killed bacteria or conditioned medium (CM) and analysed monocytes for signs of activation by flow cytometry. The mechanisms were explored using anti-CD14- or anti-TLR2 antibodies. Finally, to investigate potential pathogen-associated molecular patterns in CM, we performed mass spectrometry (MS).

Our findings revealed that *P. harei* significantly induced activation of monocytes, as evidenced by increased expression of activation markers (PDL1, HLA-DR and CD11b), phosphorylation of members of signalling pathways (NFκBp65, p38 and Akt) and production of cytokines (IL-1β, TNF, IL-6). Interestingly, *P. micra* had minimal impact on monocyte activation, and we thus focused on *P. harei.* Pre-treatment with anti-CD14 attenuated the activation of monocytes by heat-killed *P. harei*. Moreover, *P. harei* CM also induced clear monocyte activation, though, in contrast to heat-killed *P. harei*, this activation was dependent on TLR2 signalling. MS analysis of CM primarily identified four proteins possibly associated with monocyte activation. Finally, we also explored neutrophil activation and found a similar activation pattern to that of monocytes, suggesting that our observations are not exclusive to monocytes.

Our study demonstrates that *P. harei* induce prominent activation of monocytes in blood, highlighting a pathogenic potential of these otherwise harmless perceived bacteria.

**Graphical abstract:**

**Supplementary Information:**

The online version contains supplementary material available at .10.1007/s00430-025-00859-7

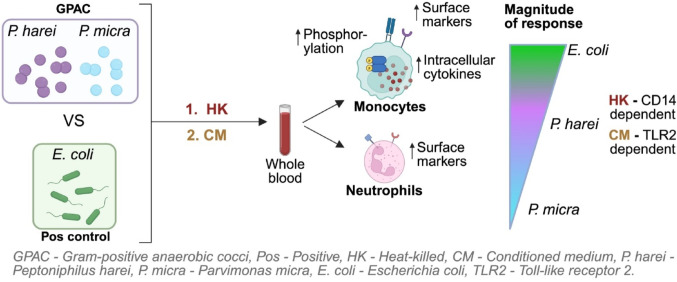

## Introduction

Bacteraemia, or the presence of bacteria in the blood stream, can cause activation of leukocytes, resulting in processes such as cytokine production which ultimately can lead to sepsis. Sepsis, a life-threatening multi-organ dysfunction, is an overreaction or failure of the host’s immune system to respond to invading pathogens [1]. The excessive amount of pro-inflammatory activation can subsequently lead to plasma leakage, organ failure and death [2]. Sepsis can be caused by a wide range of both Gram-negative and Gram-positive bacteria. One major bacterial pathogen causing sepsis or bacteraemia is *E. coli* [3], a facultative anaerobic Gram-negative bacterium. Additionally, Gram-positive bacteria account for around 50% of the cases, due to bacteria such as *S. aureus* and *S. pyogenes*. Collectively, the bacteria employ a range of virulence factors which mediates their pathogenicity, resulting in systemic inflammation [4–6].

Less well-studied bacteria in the area of sepsis/bacteriaemia include Gram-positive anaerobic cocci (GPAC). Though GPAC are considered commensals and bacteriaemia is uncommon compared to other species (e.g. *S. aureus* and *E. coli*), a retrospective study suggested an incidence of 3.4 cases of GPAC bacteraemia in 100,000 persons/year [7]. Furthermore, GPAC display an increased antibiotic resistance [8]. Despite a heightened awareness of the presence of anaerobic pathogens in septic conditions and other detrimental conditions, these bacteria are generally overlooked [9, 10]. Several reasons exist, such as the polymicrobial nature of GPAC infections and difficulties in culture and classification [11]. Indeed, even though they are primarily involved in polymicrobial infections, they may still contribute to the pathogenesis, as failure to pay attention to anaerobic culture results can have adverse effects for patients [12]. However, the presence of GPAC in bacteriaemia is becoming increasingly recognized, with two of the most common identified species being *Parvimonas micra* and *Peptoniphilus harei* [13]. Even though they are among the most commonly identified GPAC species, few studies have investigated their role in driving inflammation or direct host interactions.

Bacteriaemia and sepsis cause systemic inflammation primarily driven by components of the innate immune system. At the cellular level, myeloid cells, such as monocytes, play a pivotal role in driving this inflammation. These cells are activated, e.g. through toll-like receptor (TLR) signalling, resulting in the expression of activation markers, the production of inflammatory cytokines such as IL-6 and TNF and functional alterations [14]. Importantly, monocyte activation also has implications in long-term complications of sepsis, as the monocytes are believed to transit into an immunosuppressive state, contributing to the immuno-paralysis and long-term sequalae observed in post-sepsis patients [14, 15]. Moreover, the process and degree of monocyte activation likely varies depending on the underlying bacteria and its pathogen associated molecular patterns (PAMPs; e.g. *S. aureus* vs *S. pyogenes*) [16]. The most striking example is Gram-positive vs Gram-negative bacteria, which induce activation through different mechanisms (e.g. TLR2 vs TLR4) and subsequent downstream signalling pathways [17]. Collectively, this highlights that different bacteria induce a varying degree of monocyte activation through diverse mechanisms, underlying the need to characterize the immune response to specific bacteria.

Therefore, there is a dire need to map the monocyte response induced by GPAC. Understanding the underlying mechanisms and the degree of activation induced by GPAC is crucial in order to understand the pathogenesis and potential treatment targets. Thus, in the present exploratory study, we aimed to compare two of the most common GPAC species (*P. harei* and *P. micra*) with common causes of bacteriaemia and sepsis (e.g. *E. coli, S. pyogenes and S. aureus*), in terms of their ability to induce activation of monocytes in blood.

## Material and Methods

### Bacterial strains

*E. coli* ATCC strain 25,922 and *S. aureus* subsp. *aureus* Rosenbach ATCC 29213 were kindly provided by Prof. Artur Schmidtchen, Lund University. *S. pyogenes* strain AP1 (40/58) serotype M1 (WHO Collaborating Centre for references and research on Streptococci, Institute of Hygiene and Epidemiology, Prague, Czechia) was kindly provided by Prof. Lars Björck. *E. coli*, *S. aureus* and *S. pyogenes* were grown in TH medium until 0.5 OD. *P. harei* 5984 and *P. micra* 8984 were isolated from blood by Dr. Erik Senneby at Lund University Hospital. Both GPAC strains were identified with MALDI-TOF MS (score value > 2.0). For the cultivation, 40 µl of the bacterial stock was inoculated into 10 mL fresh TH with 0.5% Tween-80 broth and grown for 4 days in an anaerobic chamber in glass tubes. For the experiments, all bacterial strains were spun for 10 min at 3000 g, growth medium was collected, and the bacterial pellet was adjusted in PBS to 2*10^9^ cfu/ml. The conditioned medium (CM) was filtered through a 0.2 µm filter, concentrated 8X using Amicon® Ultra Centrifugal Filter, 3 kDa MWCO (Millipore) and then stored at −20 °C. For heat-inactivation of the bacteria, adjusted pellets were incubated for 20 min at 80 °C and subsequently stored at −20 °C until further usage.

### Activation of whole blood

Blood was collected in heparin tubes (BD) from healthy donors upon informed consent according to the ethical permit 2008/657 (Lund University) and used immediately. 100 µl of blood was diluted with 100 µl of RPMI-1640 medium (Sigma) in polypropylene FACS tubes (Falcon). For heat-killed and live bacteria, diluted blood was stimulated with 1*10^6^ cfu/ml of bacteria. Diluted blood without bacteria served as a negative control. For stimulation with bacterial supernatants, diluted blood was stimulated with 1 µl of the bacteria’s respective growth medium. Growth medium without bacteria served as negative control. When indicated, the blood was pre-treated for 1 h at 37 °C with a final concentration of 125 µg/ml of anti-CD14 (Atibuclimab, MedChemExpress) or 10 µg/ml of anti-TLR2 (Invivogen) antibodies before activation as described above.

### Analysis of surface marker expression

Blood was prepared as described above and incubated at 37 °C, 5% CO_2_, for 4 h. The blood was briefly vortex once every hour. For the last 20 min, the cells were stained with 50 µl brilliant violet staining buffer (BD) and 50 µl PBS with: 1:60 anti-CD66b FITC (clone: G10F5, BD), 1:100 anti-CD16 PerCP Cy5.5, (clone: 3G8 Biolegend), 1:100 anti-CD11b AF700 (clone: ICRF44, BD), 1:150 anti-CD14 Alexa fluor 700 (clone: 63D3, Biolegend), 1:150 anti-HLA-DR APC-H7 (clone: G46-6, BD), 1:150 anti-PDL1 Bv421 (clone: MIH1, BD), 1:125 anti-CD3 BV786 (clone: UCHT1, BD), 1:300 anti-CD56 BV786 (clone: NCAM 16.2, BD) and 1:300 anti-CD19-BV786 (clone: HIB19, BD). Next, the blood was fixed and lysed by addition of 1 ml of lyse/fix solution (BD), followed by a 10 min incubation, 37 °C. Next, the cells were washed twice with PBS and analyzed by flow cytometry (CytoFLEX). The Gating strategy can be found in Supplemental Fig. 1A and Supplemental Fig. 2A.

**Fig. 1:**
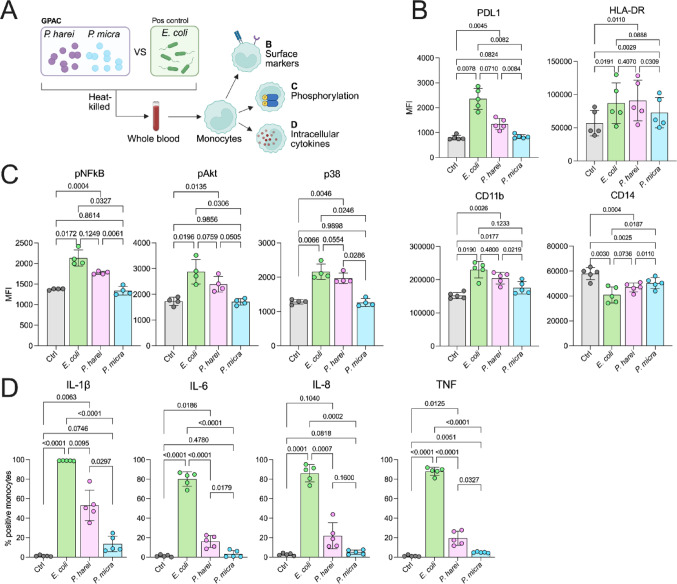
***P. harei induces prominent monocyte activation in whole blood.*** **a** Schematic overview of the experimental setup. Monocyte activation was investigated in three ways: (B) Activation (surface) markers, (C) phosphorylation of signaling pathways or (D) intracellular production of cytokines. Heat-inactivated *E. coli*, *P. harei*, and *P. micra* were used to stimulate whole blood from healthy donors for (B and D) 4 h or 10 min (C), followed by analysis by flow cytometry. **b** To analyze monocyte activation at the surface level, the expression of four activation markers was investigated (n = 5 donors). **c** Shows data on phosphorylation of three members of important signaling pathways in innate immunity (n = 4). **d** Monocytes were pre-treated with GolgiPlug before activation. The data depicts inflammatory cytokines as the percentage of positive monocytes (n = 5). The data is presented as median fluorescence intensity (MFI) or percent positive monocytes. Data is presented as mean with SD and was analysed with repeated measures one-way ANOVA with Tukey’s multiple comparisons test. The data was generated from two independent experiments. *P* < 0.05 was considered statistically significant*. *Panel A was created using Biorender.com (2025, https://BioRender.com/yufponw).* Ctrl – Control, MFI – Median fluorescence intensity. *

**Fig. 2:**
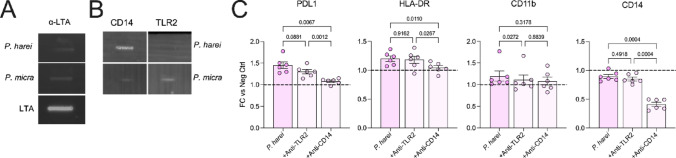
**Heat-inactivated *P. harei*-mediated activation is mainly dependent on CD14 signaling.** **a** The presence of lipoteichoic acid (LTA) was investigated in *P. harei* and *P. micra*. 150 µl whole bacteria were subjected to PVDF membrane and then incubated for 1 h with respective antibody or labelled protein. Heat-inactivated *P. harei* and *P. micra* were loaded and incubated with anti-LTA. 0.5 µg/ml LTA served as positive control. **b** PVDF membrane with heat-inactivated bacteria was incubated with fluorescent-labelled CD14 or TLR2 for 1 h. **c** Whole blood was treated with anti-CD14 (125 µg/ml) or anti-TLR2 (10 µg/ml) for 1 h followed by addition of heat-killed *P. harei*. The data shows the expression of four activation markers in monocytes. Each dot represents a unique donor (n = 6), and data is displayed as fold change to unstimulated control as mean with SD. The data was generated from three independent experiments. Data was analyzed with repeated measures one-way ANOVA with Tukey’s multiple comparisons test. *P* < 0.05 was considered statistically significant.

### Intracellular cytokine production

Blood was diluted as described above. Next, 0.5 µl Golgiplug (BD) was added, followed by bacteria, and the blood was incubated at 37 °C, 5% CO_2_, for 4 h. The blood was briefly vortex once every hour. For the last 15 min, the blood was stained with 1:150 anti-CD14 Alexa fluor 700 (clone: 63D3, Biolegend), 1:150 anti-CD3 Bv510 (clone: UCHT-1, BD) and 1:250 anti-CD19 BV786 (clone: HIB19, BD). Next, the samples were lysed and fixed for 10 min (lyse/fix buffer, BD), at 37 °C, followed by a wash with PBS. The cells were subsequently permeabilized with 1 ml perm/wash buffer (BD) for 10 min. Next, the tubes were centrifuged, and the cells were stained with anti-IL1ß AF647 (clone JK1B-1, Biolegend), anti-IL-6 PE/Cy7 (cloneMQ2-13A5, Biolegend), anti-IL-8 AF488 (clone E8N1, Biolegend) and anti-TNF BV650 (cloneMAb11, BD), all diluted 1:75, for 25 min at room temperature. The cells were subsequently washed and analyzed by flow cytometry (CytoFLEX). The Gating strategy can be found in Supplemental Fig. 1B and Supplemental Fig. 2B.

### Phosphorylation analysis

Blood was prepared as described above. Following addition of bacteria, the tubes were incubated for 10 min, 37 °C, 5% CO_2_, with surface antibodies: anti-CD66b AF700 (clone G10F5, 1:125 Biolegend) and anti-CD14 BV421 (clone HCD14, 1:150, Biolegend). The incubation was followed by immediate addition of 1 ml of lyse/fix buffer (BD) and a subsequent 10 min incubation at 37 °C. Next, the tubes were centrifuged and washed once with PBS. Permeabilization was performed using ice-cold Perm III buffer (BD) for 30 min on ice. The cells were next washed twice with PBS and stained with anti-Akt (pS473) PE (clone: M89-61, BD), anti-NFκB p65 (pS529) AF488 (clone: K10-895, BD) and anti-p38 (pT180/pY182) PerCP Cy5.5 (clone 38/p38, BD), all diluted 1:25 but Akt, which was diluted 1:100, for 25 min, room temperature. The cells were washed a final time and analyzed by flow cytometry (CytoFLEX). Gating strategy can be found in Supplemental Fig. 1C and Supplemental Fig. 2C.

### Slot blot binding assay

A total of 150 µl 2*10^8^ cfu/ml heat-inactivated bacteria [18, 19] (*P. harei* or *P. micra*) or 0.5 µg/ml LTA (positive control; InvivoGene) were loaded onto a methanol-activated Immobilon-P membrane (Merck). Membranes were blocked for 1 h at RT with 3% BSA in PBS-0.05% Tween (PBS-T). To investigate if the GPAC species carry LTA on the surface, the membrane was incubated with anti-Lipoteichoic Acid (Bioscience) diluted 1:500 for 1 h at RT. The membrane was subsequently washed thrice with PBS-T. Next, the membrane was incubated with goat anti-mouse A488 (Invitrogen) and the signal was detected using the ChemiDoc Imaging System from Biorad. To analyse potential interaction of the bacteria with CD14 or TLR2, recombinant proteins (2616-TR and 383-CD/CF, both Biotechne) were labelled with Alexa Fluor™ 488 NHS Ester (Invitrogen) or Alexa Fluor™ 633 NHS Ester (Invitrogen), respectively. After blocking with 3% BSA, the Immobilon-P membrane was incubated with the labelled proteins (diluted 1:50) for 1 h under rotation in dark. The membrane was washed thrice, and the fluorescent signal was detected with the ChemiDoc Imaging system (Biorad).

### Investigating potential secreted PAMPs in bacterial supernatants

For preliminary analysis of potentially secreted PAMPs of *P. harei* and *P. micra*, the CM was boiled for 5 min at 95 °C with 4X Laemmli buffer. TH-Tween broth without bacterial culture served as negative control for the GPAC strains. *E. coli* conditioned medium (CM) and TH broth were loaded as internal controls. As described above, CM was filtered through a 0.2 µm filter to ensure complete removal of cellular contamination. Ten µl samples were loaded on 4–20% TGX gel (Biorad) and run for 30 min at 150 V. Protein bands were visualized with GelCode™ Blue Safe Protein Stain (Thermo) as recommended by the manufacturers and images were obtained using a Gel Doc Imager (Bio-Rad). To further investigate the conditioned medium of *P. harei*, 15 µl material were loaded on a 4–20% TGX gel (Biorad) and run for 50 min at 150 V. The three most prominent bands of the *P. harei* CM were excised and prepared for proteomics mass spectrometry as described [20]. The dried peptides were reconstituted in 10 µl of 0.2% formic acid, 0.2% acetonitrile prior to MS analysis.

### Liquid Chromatography Tandem Mass Spectrometry (LC–MS/MS)

For LC–MS/MS analysis, 1 μl of reconstituted peptides were analysed on an Orbitrap Eclipse mass spectrometer connected to an ultra‐high‐performance liquid chromatography Dionex Ultra300 system (both Thermo Scientific) using data dependent acquisition (DDA). The peptides were loaded and concentrated on an Acclaim PepMap 100 C18 precolumn (75 μm × 2 cm) and separated on an Acclaim PepMap RSLC column (75 μm × 25 cm, nanoViper, C18, 2 μm, 100 Å; both columns Thermo Scientific), at a column temperature of 45 °C and a maximum pressure of 900 bar. A linear gradient of 3–25 of 80% acetonitrile in aqueous 0.1% formic acid was run for 50 min followed by a linear gradient of 25–45 of 80% acetonitrile in aqueous 0.1% formic acid for 10 min. One full MS scan (resolution 120,000; mass range of 350–1,400 m/z) was followed by MS/MS scans (resolution 15,000) with a 3 s cycle time. Precursors with a charge state of 2–6 were included. The precursor ions were isolated with 1.6 m/*z* isolation window and fragmented using higher-energy collisional-induced dissociation (HCD) at a normalized collision energy (NCE) of 30. The dynamic exclusion was set to 30 s and the mass tolerance window to 10 ppm.

### Data Analysis

Raw DDA data were analysed in Proteome Discoverer 2.5 against the *P. harei* Uniprot reference proteome (Proteome ID: UP000003705) strain ACS-146-V-Sch2b containing 1742 entries. Fully tryptic digestion was used, allowing for two missed cleavages. Carbamidomethylation (C) was set to static, and protein N-terminal acetylation and oxidation (M) to variable modifications. The mass tolerance for precursor ions was set to 10 ppm and for fragment ions to 0.02 Da. The protein false discovery rate (FDR) was set to 1%. Proteins identified by two or more unique peptides were considered relevant as well as those identified with high confidence, whereas others were discarded. The mass spectrometry data were deposited in the ProteomeXchange [21, 22] consortium via the MassIVE partner repository (https://massive.ucsd.edu/) with the dataset identifier PXD054705. Dataset access for reviewers – Username: MSV000095555_reviewer, Password: P*h4r3!.

### Statistical analysis

This study was exploratory in nature, aiming to identify potential trends in monocyte activation in response to bacterial exposure. Data were analysed by GraphPad Prism v9.5 (GraphPad Software). Flow cytometry data was analysed using the CytExpert software (v2.3) or Kaluza (v2.1), both Beckman Coulter. Data is shown as mean ± SD if not otherwise stated. Data comparing different bacteria were tested using repeated measures one-way ANOVA with Tukey’s post comparison test for multiple testing. A p-value of < 0.05 was considered statistically significant. Proteins identified by MS were analysed using STRING [23], with an interaction score 0.7 (high confidence). FDR is displayed as FDR (-log10).

## Results

### *P. harei* is a potent inducer of monocyte activation in blood

First, we set out to explore the potential role of Gram-positive anaerobic cocci (GPAC) to induce monocyte activation in blood, using two of the most common bacteriaemia- and sepsis associated GPAC species, *P. harei* and *P. micra*, as examples [13]. In addition, *E. coli* was used as a positive control, given its established role in blood born infections. Specifically, we stimulated whole blood with heat-inactivated bacteria and analysed the monocytes for activation by flow cytometry, analysing activation markers (PDL1, HLA-DR, CD11b and CD14), phosphorylation of members of common signalling pathways (Akt, p38 and NFκB) and intracellular cytokine production (IL-1β, IL-6, IL-8 and TNF) (Fig. 1A). Representative histograms for the data underlying Fig. 1 can be found in Supplemental Fig. 2. At the surface marker level, stimulation of whole blood (n = 5) with heat-killed *P. harei* resulted in upregulation of PDL1, HLA-DR and CD11b (*p* < 0.0110) and downregulation of CD14 (p = 0.0004) (Fig. 1B). *P. micra* induced expression of HLA-DR (p = 0.0029) and CD11b (p = 0.0177) but not PDL1 (p = 0.0824) and downregulation of CD14 (p = 0.0025). Interestingly, *P. harei* induced a higher expression of PDL1, HLA-DR and CD11b compared to *P. micra* (*p* < 0.0309), and a more prominent downregulation of CD14 (p = 0.0110). Finally, as expected, *E. coli* stimulation induced increased expression of PDL1, HLA-DR and CD11b (*p* < 0.0191), with a trend of higher expression than both *P. harei* and *P. micra*. Additionally, the downregulation of CD14 by *E. coli* was more apparent than the downregulation induced by *P. harei* (p = 0.0736) and *P. micra* (p = 0.0187). At the phosphorylation level, both *P. harei* (*p* < 0.0135) and *E. coli* (*p* < 0.0196) induced significantly more phosphorylation of all analysed markers compared to the unstimulated control (n = 4) (Fig. 1C). In contrast, *P. micra* did not induce markable phosphorylation. Accordingly, both *P. harei* and *E. coli* showed higher levels compared to *P. micra* (*p* < 0.0505), whilst there were only minor differences between *E. coli* and *P. harei*. Finally, at the cytokine level (n = 5), *E. coli* was clearly the most potent inducer of all measured cytokines (*p* < 0.0001), followed by *P. harei* which induced significant levels of IL-1β (p = 0.0063), IL-6 (p = 0.0186) and TNF (p = 0.0125), whilst *P. micra* only induced significant levels of TNF (p = 0.0051) (Fig. 1D). *E. coli* was accordingly more potent than both *P. harei* and *P. micra* (*p* < 0.0095), whilst *P. harei* induced more production of IL-1β, IL-6 and TNF (*p* < 0.0327) compared to *P. micra*. Taken together, *P. harei* (and to a much lesser extent *P. micra*) is a potent inducer of monocyte activation in whole blood.

To confirm that heat-inactivation of the bacteria did not significantly influence our results, we compared the response of live *P. harei* and *P. micra*, to investigate if the pattern of activation is similar to heat-killed bacteria using surface markers and cytokine production as readouts (n = 3, Supplemental Fig. 3). Previous studies by *de Chateau *et al. [18] and ourselves, [24] reported that heat-inactivation of bacteria facilitate handling, whilst having no effect on the bacterial surface proteins. *E. coli* was excluded from this experimental setup since its doubling time exceeds that of the GPACs by far. The difference at the surface level was less pronounced, but *P. harei* induced PDL1 expression (p = 0.0064) and *P. micra* HLA-DR (p = 0.0061, Supplemental Fig. 3A). There was a minor but statistically significant (p = 0.0417) difference in CD14 expression between *P. harei* and *P. micra*. However, differences were more pronounced at the cytokine level with marked production of IL-1β (p = 0.0029), IL-6 (p = 0.0042), IL-8 (p = 0.0040) and TNF (p = 0.0042) of *P. harei* compared to *P. micra* (Supplemental Fig. 3B). Hence, *P. harei* induced an overall stronger response compared to *P. micra*, in line with was observed for heat-killed bacteria.

**Fig. 3:**
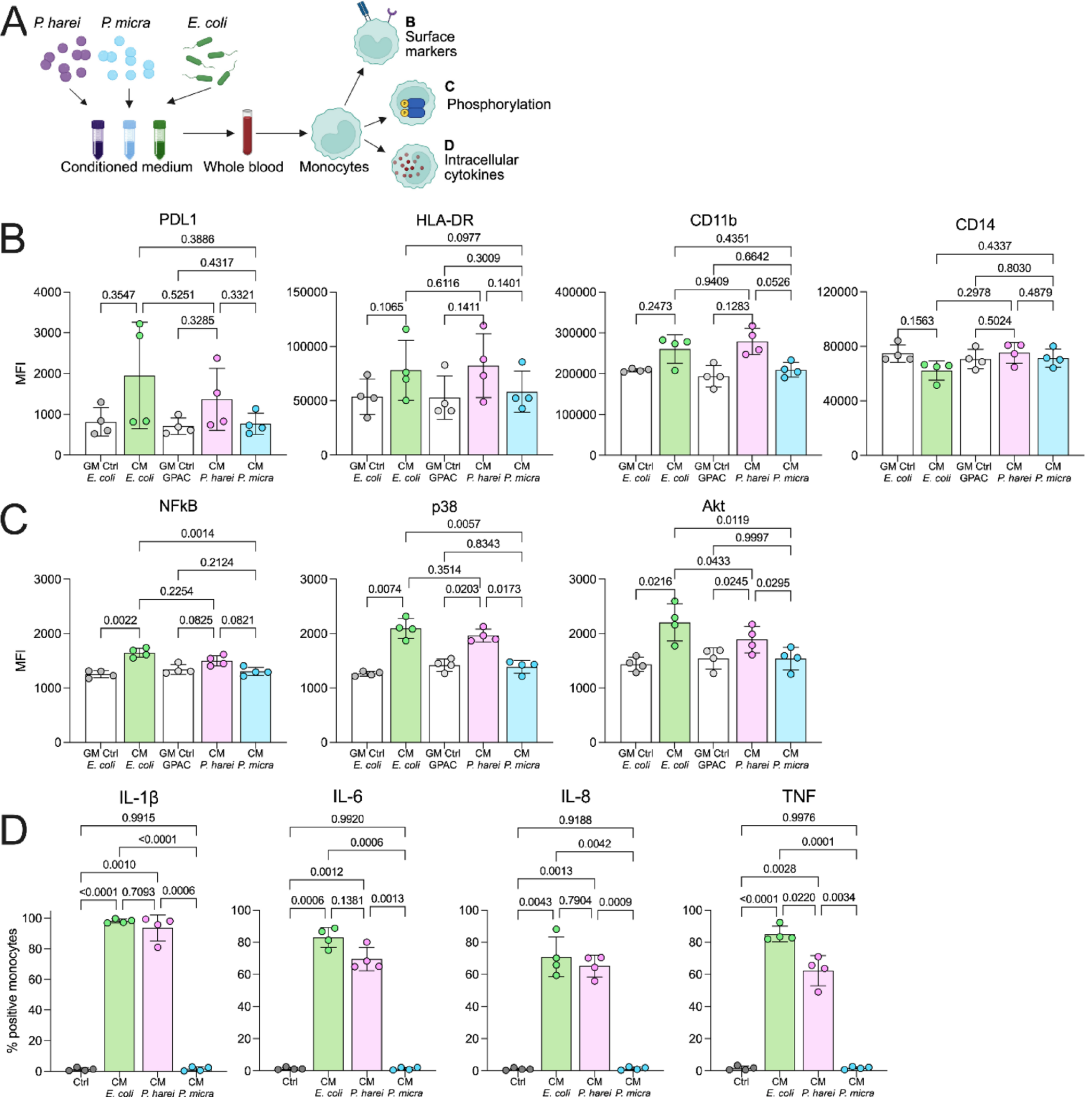
**Soluble factors of *P. harei* are potent inducers of immune cell activation in blood.** **a** Schematic outline of the experimental setup. Whole blood was stimulated with conditioned medium from *E. coli*, *P. harei*, *and P. micra* for 4 h before analysis for (B) surface marker expression, (C) phosphorylation or (D) intracellular production of cytokines by monocytes. Growth medium (GM) was used as a negative control. **b** Displays surface expression of the three markers of activation (PDL1, HLA-DR and CD11b) on monocytes, as analyzed by flow cytometry. **c** Summary data on phosphorylated NFkB, p38 and Akt in monocytes following activation with CM for 10 min. **d** Shows data on the intracellular production of cytokines in monocytes. Gates were set according to the growth medium control (without bacteria). Each dot represents a unique donor (n = 3–4), and data is displayed as mean with SD. Data was analyzed with repeated measures one-way ANOVA with Tukey’s multiple comparisons test. The data was generated from two independent experiments. *P* < 0.05 was considered statistically significant. Panel A was created using Biorender.com (2025, https://BioRender.com/v52sd2a). *Ctrl — Control, GM- growth medium, CM — conditioned medium, MFI – Median fluorescence intensity*

### Monocyte activation induced by P. harei is more pronounced compared to other Gram-positive bacteria

Next, to put our results in the context of Gram-positive bacteria, we compared the response of *P. harei* and *P. micra* to other Gram-positive bacteria (i.e. *S. aureus* and *S. pyogenes)*. At the surface marker level (n = 5), *P. harei* induced a higher degree of expression of all markers (PDL1 (*p *< 0.0299), HLA-DR (*p *< 0.0403) and CD11b (*p* < 0.0384)) compared to both *S. pyogenes* and *S. aureus* (Supplemental Fig. 4A). Additionally, *P. harei* induced downregulation of CD14 compared to *S. pyogenes* (p = 0.0007) and *S. aureus* (p = 0.0028). There were only minor differences for *P. micra*, as it induced a higher HLA-DR expression (p = 0.0051) compared to *S. aureus*, but *S. pyogenes* induced a higher PDL1 expression (p = 0.0301). At the cytokine level, there was no statistically significant differences. This could possibly be due to the small sample size (n = 3), as there was a trend of higher cytokine production of *P. harei* compared to the other bacteria (Supplemental Fig. 4B). Hence, *P. harei*, but not *P. micra*, is a potent activator of monocytes in blood compared to the other Gram-positive bacteria studied here. Therefore, we focused on the comparison to the most potent activator, *E. coli*.

**Fig. 4:**
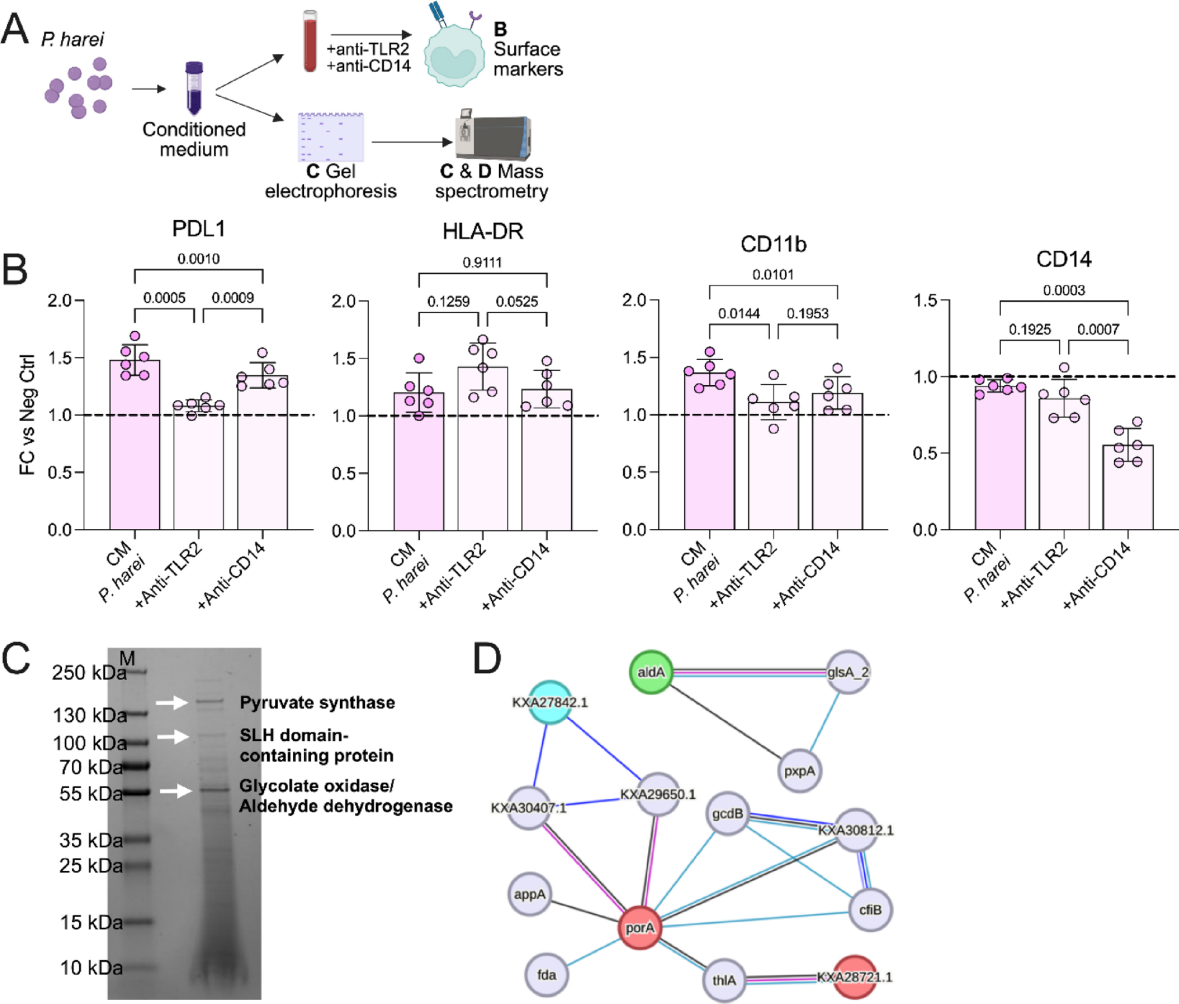
**Soluble factors of *P. harei* induce monocyte activation primarily via TLR2.** **a** Overview of the experimental setup were the mechanism of monocytes activation by conditioned medium of *P. harei*, was investigates, and potential underlying candidates were explored via gel electrophoresis and mass spectrometry analysis of prominent bands. **b** Whole blood was pre-treated with anti-TLR2 (10 µg/ml) or anti-CD14 (125 µg/ml) for 1 h followed by addition of supernatants of *P. harei*. Shows expression of surface markers in monocytes following activation with conditioned medium (CM). Growth medium without bacteria served as negative control and data is presented as fold change (FC) to the negative control*.* Each dot represents a unique donor (n = 6), and data is displayed as mean with SD. Data was analyzed with repeated measures one-way ANOVA with Tukey’s multiple comparisons test. The data was generated from three independent experiments. Next, *P. harei* CM was analysed by SDS PAGE and MS. **c** Gel electrophoresis of *P. harei* CM. Bands marked with 1–3 were excised and analysed after in-gel digestion by MS and the main identified proteins are highlighted. **d** STRING analysis of protein networks of the most abundant proteins (coloured balls) found in *P. harei* CM experimentally determined (magenta lines), database annotated (cyan lines), co-expression (black lines) and gene co-occurrence (dark blue lines) < 10 interactor, confidence 0.500. P < 0.05 was considered statistically significant. Panel A was created using Biorender.com (2025, https://BioRender.com/8lmylbo). *MFI — Median fluorescence intensity, CM — Conditioned medium, MS — Mass spectrometry*

### P. harei activation of monocytes is mainly dependent CD14 signaling

Next, we aimed to investigate the mechanism of monocyte activation by *P. harei*. Several surface proteins of Gram-positive bacteria are known to elicit immune responses, especially lipoteichoic acid (LTA; [25]). Thus, we investigated the expression of LTA in *P. micra* and *P. harei,* to check differences in expression. While the previous experiments showed differences between *P. micra* and *P. harei* in their ability to trigger immune responses, expression of LTA appeared equal between the species (Fig. 2A). Hence, we instead checked for differences in binding of the bacteria to two different receptors. TLR2 is associated with Gram-positive factor recognition [26, 27] and full TLR2 activation depends on the presence of GPI-anchored CD14 as a co-receptor [28–31]. We therefore tested the ability of *P. harei* and *P. micra* to bind to either receptor. A strong fluorescent signal was detected for the interaction with *P. harei* with CD14, while no interaction was observed with TLR2 (Fig. 2B**, upper panel**). Only minor signal was detected for the interaction of *P. micra* with CD14, as well as for TLR2 (in contrast to *P. harei*) (Fig. 2B**, lower panel**). Thus, the pronounced activation of monocytes by *P. harei* compared to *P. micra* could be due to more prominent CD14 binding.

To study this, we pre-incubated monocytes (n = 6) with inhibitory antibodies against either TLR2 or CD14, before stimulation with heat-killed *P. harei*. While only slight changes were detected in the expression patterns with anti-TLR2 pre-treatment, anti-CD14 pre-treatment prevented the increase in PDL1 (p = 0.0067) and HLA-DR (p = 0.0110) by *P. harei* (Fig. 2C). The trend was similar for CD11b although it was not markedly induced in this experiment. In samples treated with anti-CD14, there was a marked reduction of CD14 expression. This is possibly due to downregulation or recycling of the CD14 receptor as a result of the pre-treatment. Still, we hypothesise that the activation of monocytes in whole blood by *P. harei* is mainly dependent on CD14 signalling.

### Soluble mediators of P. harei are comparable to those of E. coli at inducing monocyte activation in blood

Factors secreted by the bacteria may be a crucial source of virulence and monocyte activation. To investigate a potential effect of soluble mediators, we incubated whole blood with conditioned medium (CM) of the bacteria (Fig. 3A). At the surface level, there was a trend of higher expression of PDL1, CD11b and HLA-DR in monocytes although it did not reach statistical significance, likely due to the data spread and the small sample size (n = 4) (Fig. 3B). Moreover, there was a non-significant trend of lower CD14 expression in *E. coli* treated samples but not in GPAC treated samples. More strikingly, at the phosphorylation level, both CM from *E. coli* and *P. harei* induced phosphorylation of NFκB (*p < *0.0272), Akt (*p *< 0.0055) and p38 (*p < *0.0084 (Fig. 3C). Compared to *E. coli*, there was a trend towards weaker signal in *P. harei* CM which was significant for Akt (p = 0.0433). No phosphorylation was induced by *P. micra* CM. Finally, there was a major induction of intracellular cytokine production of IL-1β (*p *< 0.001), IL-6 (*p* < 0.0012), IL-8 (*p* < 0.0043) and TNF (*p* < 0.0028) in monocytes by *E. coli* and *P. harei*, but not *P. micra* (Fig. 3D). Interestingly, there were no statistical differences in cytokine production between *P. harei* and *E. coli*. Taken together, soluble mediators by *P. harei* potently induce monocyte activation, comparable in several of the markers to that of *E. coli*.

### Monocyte activation by P. harei conditioned medium is dependent on TLR2 signalling

Since we observed a positive effect of pre-treating blood with anti-CD14 prior to stimulation with heat-inactivated *P. harei*, we hypothesised that inhibition of CD14 could also diminish the host responses mediated by the bacterial supernatant (Fig. 4A). However, anti-TLR2 pre-treatment blocked the upregulation PDL1 (p = 0.0005) and CD11b (p = 0.0144) (Fig. 4B). An effect of anti-CD14 could still be observed for PDL1 (p = 0.001) and CD11b (p = 0.0101), although anti-TLR2 inhibition was more potent for PDL1 (p = 0.009). Again, as with heat-killed bacteria, samples pre-treated with anti-CD14 had markedly reduced CD14 expression. Thus, in contrast to the CD14-dependent activation by heat-killed *P. harei*, activation by *P. harei* CM is dependent on TLR2 signalling, suggesting different mechanisms of activation depending on interaction with surface bound- or soluble factors.

### Identification of possible soluble factors of P. harei involved in monocyte activation

Next, we aimed to characterize potential secreted candidates responsible for the observed activation. Gel electrophoresis analysis of the CM identified several bands that were exclusively present in the CM derived from *P. harei* (Supplemental Fig. 5), while no protein bands were detected in the CM of either *P. micra, E. coli* or their respective media alone.

**Fig. 5:**
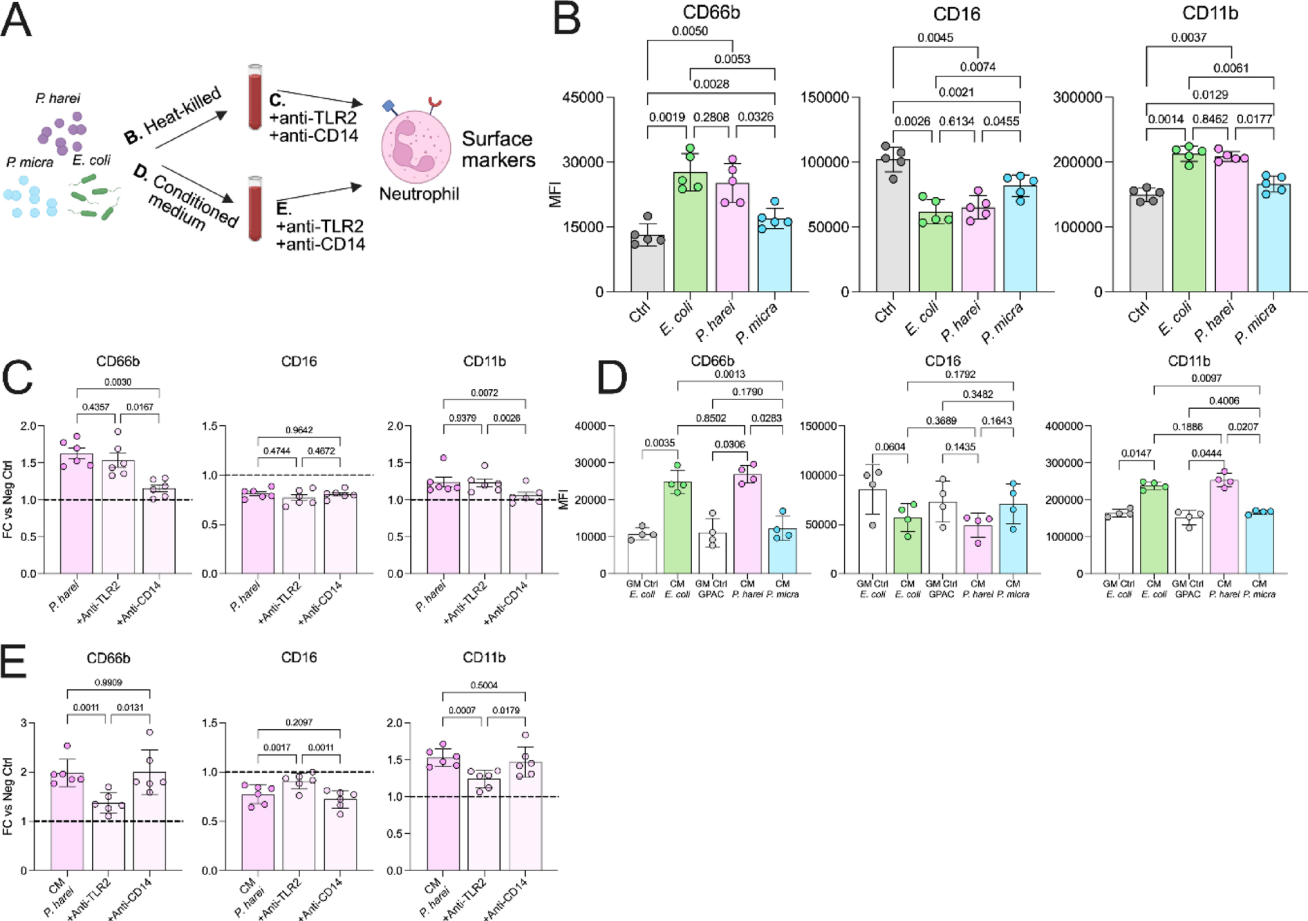
***P. harei *is a potent activator of neutrophils in blood through similar mechanisms as monocytes**. **a** To generalize our findings, we also investigated the ability of *P. harei*, *P. micra* and *E. coli* to induce neutrophil activation (exemplified by surface marker expression of three activation markers). Heat-inactivated as well as conditioned medium was used to induce activation, and the effects of anti-TLR2 and anti-CD14 were studied using *P. harei* (C and E). **b** Whole blood was incubated with heat-inactivated bacteria for 4 h before the analysis of activation markers by flow cytometry (n = 5). The data is presented as median fluorescence intensity (MFI) and depicted as mean with SD. **c** The blood was pre-treated with anti-CD14 (125 µg/ml) or anti-TLR2 (10 µg/ml) for 1 h before the addition of the bacteria. data is presented as fold change (FC) to the negative control*.* Each dot represents a unique donor (n = 6), and data is displayed as mean with SD. (**d**–**e**) Conditioned medium was used instead of heat-inactivated bacteria to induce activation in n = 4–6 donors. Data was analyzed with repeated measures one-way ANOVA with Tukey’s multiple comparisons test. The data in (B) and (D) were generated from two independent experiments and for (C) and (E) it was generated from three independent experiments. *P* < 0.05 was considered statistically significant. Panel A was created using Biorender.com (2025, https://BioRender.com/i8bjppr).

To assess whether the detected bands in *P. harei* CM correlate with known PAMPs, we analysed the CM by SDS PAGE and proteomics mass spectrometry. The three most prominent bands (at > 130 kDa, 110 kDa and 55 kDa; Fig. 4C), were further processed by in-gel digestion mass spectrometric analysis (Supplemental Table 1). These bands were identified based on respective abundances as pyruvate synthase (band 1 at > 130 kDa; calculated molecular weight (MW) 129.6 kDa, Uniprot ID: E4KZ56), SLH-domain-containing protein (band 2 at 110 kDa; calculated MW 99.9 kDa, Uniprot ID: E4KY84) and as putative glycolate oxidase (Uniprot ID: E4L019) or aldehyde dehydrogenase B (Uniprot ID: E4KZR7) (band 3 at 55 kDa; calculated MWs 51.8 and 53.8 kDa, respectively) (Fig. 4C). Whereas the pyruvate synthase, glycolate oxidase and aldehyde dehydrogenase are involved in various biochemical processes, SLH domain-containing proteins have been shown to non-covalently anchor several bacterial proteins to the cell surface [32]; often, proteins that associated with the virulence and survival of various pathogens ([33–35]). STRING network analysis of protein interactions (Fig. 4D) revealed that the pyruvate synthase, the putative glycolate oxidase and the aldehyde dehydrogenase interact with proteins involved in bacterial respiration, ion-translocation, fatty acid synthesis and carbohydrate degradation; suggestive of virulence attribute [36, 37]. Taken together, these data highlight several candidates released by *P. harei* that could be responsible for the observed activation of monocytes in blood.

### Immune cell activation by P. harei is not unique to monocytes

To generalize our findings, we next analysed neutrophils in blood (n = 4–6) for activation by using three surface markers (CD66b, CD16 and CD11b) by flow cytometry (Fig. 5A). As with the monocytes, heat-killed *P. harei* induced activation of neutrophils, evidenced by increased expression of CD66b and CD11b (*p* < 0.0050), and a downregulation of CD16 (p = 0.0045, Fig. 5B)*. P. micra* also induced activation of neutrophils (*p* < 0.0129). The magnitude of the response of *P. harei* was greater than that of *P. micra* (*p* < 0.0455) and comparable to *E. coli* (Fig. 5B). Blood pre-treated with anti-CD14 prevented the upregulation of CD66b (p = 0.0030) and CD11b (p = 0.0072) but did not prevent the CD16 downregulation induced by *P. harei* (Fig. 5C). Notably, there was no statistical effect of anti-TLR2 pre-treatment.

Using conditioned medium (CM), *P. harei* induced upregulation of CD66b (p = 0.0306) and CD11b (p = 0.0444), and downregulation of CD16, although it did not reach statistical significance (Fig. 5D). Interestingly, in contrast to the heat-killed bacteria, there was no activation induced by *P. micra*. Moreover, the activation induced by *E. coli* was comparable to that of P*. harei*. However, in contrast to the whole bacteria, we found that the activation induced by *P. harei* CM was distinctly inhibited by pre-treatment with anti-TLR2 (*p* < 0.0017, Fig. 5E) in contrast to anti-CD14. Indeed, there was no effect of anti-CD14. Taken together, these results suggest that the activation induced by *P. harei* is not unique to monocytes, highlighting its potential in inducing inflammation and immune cell activation in blood.

## Discussion

Gram-positive anaerobic cocci (GPAC) are considered as a part of the normal microbiota, although their involvement in bacteriaemia and sepsis is becoming increasingly acknowledged. Monocytes are known for their role in driving inflammation in sepsis, though their response to GPAC is not well described. Thus, we aimed to investigate the ability of two of the most commonly isolated GPAC species from blood, *P. harei* and *P. micra*, to induce monocyte activation. We observed that both heat-killed bacteria and conditioned medium (CM) of *P. harei*, and to a lesser degree *P. micra*, are potent inducers of monocyte activation in whole blood, as evidenced by surface marker expression, phosphorylation of signalling pathways and cytokine production. Importantly, the activation induced by *P. harei* was more pronounced than that of other more common Gram-positive bacteria, i.e. *S. pyogenes* and *S. aureus*, and was comparable to the response of *E. coli* in some of the experiments. The apparent inability of *S. aureus* (as well as *S. pyogenes*) to activate monocytes in our experimental setting was surprising since these bacteria are known as potent activators [38], however used concentration, time points or strain might have played a role in this. Interestingly, at the surface level, the response to heat-killed bacteria was mainly CD14-dependent, while the response to CM was mainly TLR2-dependent. Moreover, several candidates were identified in CM through mass spectrometry. Finally, to generalize our findings, we also investigated neutrophil activation and found a similar pattern of activation to that of monocytes. Thus, our results highlight an underappreciated ability of GPAC species, such as *P. harei,* in stimulating immune cell activation, in some regards comparable to potent pathogens associated with sepsis, such as *E. coli*.

In this study, we limited our focus to monocytes and, to some extent, neutrophils. Monocytes have well described roles in infections and sepsis, where their excessive response is known to contribute to disease through various mechanisms involving cytokine production and impaired clearance [15, 39, 40]. Specifically, they produce an excessive amount of cytokines, sustaining the vicious circle of inflammation which contributes to the cytokine storm [41, 42]. This has been investigated using some of the more common pathogens associated with sepsis, such as *E. coli*. GPAC are not commonly associated with bacteriaemia and sepsis, although an incidence of 3.4/100´000 persons/year has been suggested [13]. This is partly due to difficulties arising by their slow growth and taxonomic challenges. Hence, knowledge of their role in driving monocyte activation (and inflammation in general) is lacking. Here, we observed a prominent activation of monocytes by *P. harei*, similar in some regards to the response to *E. coli*. Notably, we could observe activation ranging from the signalling level to the production of cytokines and upregulation of surface markers, covering several aspects of monocyte activation. Interestingly, the response to *P. micra* was limited, suggesting that the activation induced by *P. harei* is not general for GPAC, and indicates that the monocyte-activating capabilities of different GPAC species varies significantly. Therefore, our data indicate that other members of GPAC should be investigated for their ability to induce inflammation. Data on the ability of other GPAC to induce inflammation are limited. Still, we have previously shown that *Finegoldia magna* induces neutrophil activation [43]. Thus, our results suggest a potent role of certain GPAC species in inducing monocyte activation and highlights a need to map the immune response to different GPAC to understand the pathogenesis and threat to the host.

Bacterial infections often trigger a multifaceted immune response involving several key pathways, e.g. TLR2 and NFκB signalling, recognizing specific PAMPs such as lipoteichoic acids (LTAs) and peptidoglycans. Here, while LTA is detected on the surface of both *P. harei* and *P. micra*, only weak to no interaction with TLR2 is observed for *P. harei*, indicating that other pathways might be involved in immune activation. This is also supported by the weak response of *P. micra* compared to *P. harei*. Our findings are in good correlation with earlier findings, describing the TLRs interact with soluble factors, rather than surface bound proteins [44] as they might be inaccessible. Indeed, we instead show a prominent interaction with CD14. Other studies have shown CD14 to be a solid interaction partner for cell-wall bound as well as soluble bacterial components, leading to downstream activation [45]. Hence, for *P. harei,* our results suggest that host immune activation of occurs via CD14 interaction. However, the signalling could also be TLR2 independent, as other commensal bacteria like *Lactobacillus* have been demonstrated to induce cytokine production in macrophages via a TLR2 independent, yet MyD88-dependent mechanism [46]. Nonetheless, our data indicate a prominent role of CD14 in *P. harei* induced activation of monocytes. Moreover, in contrast to the heat-killed bacteria, we found that CM-mediated immune cell activation appears to be TLR2. This finding suggests that soluble mediators of *P. harei* induce activation through a different mechanism than heat-killed bacteria. Still, these findings need to be further confirmed by exploring different functional aspects of monocytes besides surface markers alone. Indeed, we also analysed activation of neutrophils to investigate if the response observed in monocytes was cell specific. We found a similar pattern to that of monocytes, including the CD14/TLR2 inhibition, suggesting that this response is not monocyte specific. Importantly, both CD14 and TLR2 have been suggested for targeted therapy in an infection context [47, 48]. Thus, blocking these signalling pathways represents an attractive approach to limit excessive inflammation and organ damage.

Regarding potential PAMPs in the CM of *P. harei,* SDS-PAGE gel analysis in combination with proteomics of *P. harei* CM identified 3 prominent bands corresponding to 4 secreted proteins, pyruvate synthase, an SLH domain-containing protein, a putative glycolate oxidase and aldehyde dehydrogenase. Even though these proteins are major secreted proteins of *P. harei,* there is currently no description of their role in *P. harei* pathogenesis. Interestingly, some anaerobic cancer-associated bacterial pathogens, including *P. harei*, have been demonstrated to interfere with citrate and pyruvate metabolism pathways as well as steroid metabolism [49–52]. Additionally, SLH domain-containing protein (accession: E4KY84) is almost exclusively found in *Peptoniphilus* sp. strains. S-layer proteins in bacteria, which cover the bacterial surfaces, have been proposed as important PAMPs as well as potential therapeutic targets [32]. Therefore, we hypothesize that the secreted, metabolic proteins in the CM of *P. harei* meddle with host metabolic functions and signalling pathways [32, 36, 49–51]; thus, contribute to the overall pathogenic potential of *P. harei*. Nonetheless, we show that the CM is a potent inducer of monocyte activation. Indeed, in several experiments the response of *P. harei* CM was comparable to that of *E. coli* CM. These findings are important as they highlight the potency of secreted monocyte-activating components of *P. harei*, suggesting an additional mechanism of inducing inflammation. Still, besides the identified proteins, several other factors could be responsible for the observed activation, such as polysaccharides and lipids, that would not show up in the gel or MS (e.g. LPS). Thus, the potential role of the identified proteins in this study should be further explored in future studies.

There are several limitations to this study. First, only surface markers were used as a read-out for assessing anti-CD14/TLR2 inhibition. Given the relative minor changes in surface marker expression, the effect of CD14/TLR2 signalling needs to be confirmed using different assays in a future study. In addition, only a single concentration of each bacterial species was tested to ensure comparable activation conditions. However, using different concentrations may have revealed dose-dependent differences in monocyte responses, as some bacteria likely differ in their potency as immune activators. Moreover, only one time point was analysed, which limits our understanding of the kinetics of monocyte activation. Assessing multiple time points could provide insights into the temporal dynamics of the effect of bacteria on monocytes. Even though debris was excluded, a viability dye was not used. This could result in some few dead cells being included in our analysis. Finally, we were not able to test the potential monocyte-activating candidates identified in the conditioned medium by mass spectrometry, and we are going to pursue their involvement in a future study.

In conclusion, we have demonstrated the potential of *P. harei* to induce immune cell activation and inflammation in blood, driven by TLR2 and CD14 signalling. Our results provide novel insights into the potency of this otherwise commensal bacterium and provide targets for future targeting for therapy.

## Supplementary Information

Below is the link to the electronic supplementary material.

Supplementary Material 1

Supplementary Material 2

Supplementary Material 3

Supplementary Material 4

Supplementary Material 5

Supplementary Material 6

## Data Availability

The datasets generated and/or analysed during the current study are available in the ProteomeXchange [21, 22] consortium via the MassIVE partner repository (https://massive.ucsd.edu/) with the dataset identifier PXD054705. Dataset access for reviewers – Username: MSV000095555_reviewer, Password: P*h4r3!.

## References

[1] Póvoa P et al (2023) How to use biomarkers of infection or sepsis at the bedside: guide to clinicians. Intensive Care Med 49(2):142–153 36592205 10.1007/s00134-022-06956-y PMC9807102

[2] Nedeva C, Menassa J, Puthalakath H (2019) Sepsis: Inflammation Is a Necessary Evil. Front Cell Dev Biol 7:108 31281814 10.3389/fcell.2019.00108 PMC6596337

[3] Shao, Q., et al., *Escherichia coli Infection Sepsis: An Analysis of Specifically Expressed Genes and Clinical Indicators.* Diagnostics (Basel), 2023. 13(23). 10.3390/diagnostics13233542 PMC10706716 38066783

[4] Kaper JB, Nataro JP, Mobley HL (2004) Pathogenic Escherichia coli. Nat Rev Microbiol 2(2):123–140 15040260 10.1038/nrmicro818

[5] Foster-Nyarko, E. and M.J. Pallen, *The microbial ecology of Escherichia coli in the vertebrate gut.* FEMS Microbiol Rev, 2022. 46(3). 10.1093/femsre/fuac008 PMC9075585 35134909

[6] Cheung GYC, Bae JS, Otto M (2021) Pathogenicity and virulence of Staphylococcus aureus. Virulence 12(1):547–569 33522395 10.1080/21505594.2021.1878688 PMC7872022

[7] Badri M et al (2019) Clinical and microbiological features of bacteraemia with Gram-positive anaerobic cocci: a population-based retrospective study. Clin Microbiol Infect 25(6):760.e1-760.e6 30217761 10.1016/j.cmi.2018.09.001

[8] Di Bella S et al (2022) Anaerobic bloodstream infections in Italy (ITANAEROBY): A 5-year retrospective nationwide survey. Anaerobe 75:102583 35568274 10.1016/j.anaerobe.2022.102583

[9] Ryan PM, Morrey BF (2021) Parvimonas micra causing native hip joint septic arthritis. Proc (Bayl Univ Med Cent) 34(4):486–488 34219932 10.1080/08998280.2021.1906827 PMC8224201

[10] Ali H, Amir W, Bolick NL (2021) An uncommon case of native joint septic arthritis by Parvimonas micra. Anaerobe 67:102315 33348083 10.1016/j.anaerobe.2020.102315

[11] Murphy EC, Frick IM (2013) Gram-positive anaerobic cocci–commensals and opportunistic pathogens. FEMS Microbiol Rev 37(4):520–553 23030831 10.1111/1574-6976.12005

[12] Salonen JH, Eerola E, Meurman O (1998) Clinical significance and outcome of anaerobic bacteremia. Clin Infect Dis 26(6):1413–1417 9636872 10.1086/516355

[13] Badri, M., et al., *Clinical and microbiological features of bacteraemia with Gram-positive anaerobic cocci: a population-based retrospective study.* Clin Microbiol Infect, 2019. 25(6) 760 e1–760 e6. 10.1016/j.cmi.2018.09.001 30217761

[14] Shalova IN et al (2015) Human monocytes undergo functional re-programming during sepsis mediated by hypoxia-inducible factor-1alpha. Immunity 42(3):484–498 25746953 10.1016/j.immuni.2015.02.001

[15] Gritte RB et al (2022) Evidence for Monocyte Reprogramming in a Long-Term Postsepsis Study. Crit Care Explor 4(8):e0734 35928539 10.1097/CCE.0000000000000734 PMC9345639

[16] Blease K et al (1999) Lipoteichoic acid inhibits lipopolysaccharide-induced adhesion molecule expression and IL-8 release in human lung microvascular endothelial cells. J Immunol 163(11):6139–6147 10570304

[17] Rabehi L et al (2001) Gram-positive and gram-negative bacteria do not trigger monocytic cytokine production through similar intracellular pathways. Infect Immun 69(7):4590–4599 11402003 10.1128/IAI.69.7.4590-4599.2001 PMC98536

[18] De Château M et al (1993) On the interaction between protein L and immunoglobulins of various mammalian species. Scand J Immunol 37(4):399–405 8469922 10.1111/j.1365-3083.1993.tb03310.x

[19] De Château M, Holst E, Björck L (1996) Protein PAB, an Albumin-binding Bacterial Surface Protein Promoting Growth and Virulence. J Biol Chem 271(43):26609–26615 8900134 10.1074/jbc.271.43.26609

[20] Shevchenko A et al (2006) In-gel digestion for mass spectrometric characterization of proteins and proteomes. Nat Protoc 1(6):2856–2860 17406544 10.1038/nprot.2006.468

[21] Deutsch EW et al (2023) The ProteomeXchange consortium at 10 years: 2023 update. Nucleic Acids Res 51(D1):D1539-d1548 36370099 10.1093/nar/gkac1040 PMC9825490

[22] Vizcaíno JA et al (2014) ProteomeXchange provides globally coordinated proteomics data submission and dissemination. Nat Biotechnol 32(3):223–226 24727771 10.1038/nbt.2839 PMC3986813

[23] Szklarczyk D et al (2023) The STRING database in 2023: protein-protein association networks and functional enrichment analyses for any sequenced genome of interest. Nucleic Acids Res 51(D1):D638-d646 36370105 10.1093/nar/gkac1000 PMC9825434

[24] Neumann A, Björck L, Frick IM (2020) Finegoldia magna, an Anaerobic Gram-Positive Bacterium of the Normal Human Microbiota, Induces Inflammation by Activating Neutrophils. Front Microbiol 11:65 32117109 10.3389/fmicb.2020.00065 PMC7025542

[25] Reichmann NT, Gründling A (2011) Location, synthesis and function of glycolipids and polyglycerolphosphate lipoteichoic acid in Gram-positive bacteria of the phylum Firmicutes. FEMS Microbiol Lett 319(2):97–105 21388439 10.1111/j.1574-6968.2011.02260.x PMC3089915

[26] Påhlman LI et al (2006) Streptococcal M protein: a multipotent and powerful inducer of inflammation. J Immunol 177(2):1221–1228 16818781 10.4049/jimmunol.177.2.1221

[27] Wu S et al (2016) Group A Streptococcus induces less p65 nuclear translocation and non-classical nuclear factor kappa B activation in macrophages, which possibly leads to a weaker inflammatory response. Int J Infect Dis 44:50–60 26854198 10.1016/j.ijid.2016.01.018

[28] Dunne DW et al (1994) The type I macrophage scavenger receptor binds togram-positive bacteria and recognizes lipoteichoic acid. Proc Natl Acad Sci 91(5):1863–1867 8127896 10.1073/pnas.91.5.1863 PMC43264

[29] Yoshimura A et al (1999) Cutting edge: recognition of Gram-positive bacterial cell wall components by the innate immune system occurs via Toll-like receptor 2. J Immunol 163(1):1–5 10384090

[30] Gupta D et al (1996) CD14 is a cell-activating receptor for bacterial peptidoglycan. J Biol Chem 271(38):23310–23316 8798531 10.1074/jbc.271.38.23310

[31] Henneke P et al (2001) Novel engagement of CD14 and multiple toll-like receptors by group B streptococci. J Immunol 167(12):7069–7076 11739528 10.4049/jimmunol.167.12.7069

[32] Mesnage S et al (2000) Bacterial SLH domain proteins are non-covalently anchored to the cell surface via a conserved mechanism involving wall polysaccharide pyruvylation. Embo j 19(17):4473–4484 10970841 10.1093/emboj/19.17.4473 PMC302060

[33] Blackler RJ et al (2018) Structural basis of cell wall anchoring by SLH domains in Paenibacillus alvei. Nat Commun 9(1):3120 30087354 10.1038/s41467-018-05471-3 PMC6081394

[34] Przepiora, T., et al., *The Periplasmic Oxidoreductase DsbA Is Required for Virulence of the Phytopathogen Dickeya solani.* Int J Mol Sci, 2022. 23(2). 10.3390/ijms23020697 PMC8775594 35054882

[35] Pumirat, P., et al., *The role of short-chain dehydrogenase/oxidoreductase, induced by salt stress, on host interaction of B. pseudomallei.* BMC Microbiology, 2014. 14(1) 1. 10.1186/1471-2180-14-1 PMC3882111 24382268

[36] Bhargavi G et al (2022) A putative short-chain dehydrogenase Rv0148 of Mycobacterium tuberculosis affects bacterial survival and virulence. Current Research in Microbial Sciences 3:100113 35243448 10.1016/j.crmicr.2022.100113 PMC8861579

[37] McFarland AP et al (2017) Sensing of Bacterial Cyclic Dinucleotides by the Oxidoreductase RECON Promotes NF-κB Activation and Shapes a Proinflammatory Antibacterial State. Immunity 46(3):433–445 28329705 10.1016/j.immuni.2017.02.014 PMC5404390

[38] Craven RR et al (2009) Staphylococcus aureus α-Hemolysin Activates the NLRP3-Inflammasome in Human and Mouse Monocytic Cells. PLoS ONE 4(10):e7446 19826485 10.1371/journal.pone.0007446 PMC2758589

[39] Frimpong A et al (2022) Cytokines as Potential Biomarkers for Differential Diagnosis of Sepsis and Other Non-Septic Disease Conditions. Front Cell Infect Microbiol 12:901433 35811678 10.3389/fcimb.2022.901433 PMC9260692

[40] Jain K et al (2023) Reconditioned monocytes are immunomodulatory and regulate inflammatory environment in sepsis. Sci Rep 13(1):14977 37696985 10.1038/s41598-023-42237-4 PMC10495550

[41] Serbina NV et al (2008) Monocyte-mediated defense against microbial pathogens. Annu Rev Immunol 26:421–452 18303997 10.1146/annurev.immunol.26.021607.090326 PMC2921669

[42] Hessle CC, Andersson B, Wold AE (2005) Gram-positive and Gram-negative bacteria elicit different patterns of pro-inflammatory cytokines in human monocytes. Cytokine 30(6):311–318 15935951 10.1016/j.cyto.2004.05.008

[43] Neumann A, Bjorck L, Frick IM (2020) Finegoldia magna, an Anaerobic Gram-Positive Bacterium of the Normal Human Microbiota, Induces Inflammation by Activating Neutrophils. Front Microbiol 11:65 32117109 10.3389/fmicb.2020.00065 PMC7025542

[44] Underhill DM, Gantner B (2004) Integration of Toll-like receptor and phagocytic signaling for tailored immunity. Microbes Infect 6(15):1368–1373 15596122 10.1016/j.micinf.2004.08.016

[45] Jack RS et al (1995) Both membrane-bound and soluble forms of CD14 bind to gram-negative bacteria. Eur J Immunol 25(5):1436–1441 7539760 10.1002/eji.1830250545

[46] Udayan S et al (2021) Macrophage cytokine responses to commensal Gram-positive Lactobacillus salivarius strains are TLR2-independent and Myd88-dependent. Sci Rep 11(1):5896 33723368 10.1038/s41598-021-85347-7 PMC7961041

[47] Thorgersen Ebbe B et al (2013) Systemic CD14 Inhibition Attenuates Organ Inflammation in Porcine Escherichia coli Sepsis. Infect Immun 81(9):3173–3181 23774598 10.1128/IAI.00390-13 PMC3754210

[48] Simpson ME, Petri WA Jr (2020) TLR2 as a Therapeutic Target in Bacterial Infection. Trends Mol Med 26(8):715–717 32563557 10.1016/j.molmed.2020.05.006 PMC7845793

[49] Li XY et al (2023) Common pathogenic bacteria-induced reprogramming of the host proteinogenic amino acids metabolism. Amino Acids 55(11):1487–1499 37814028 10.1007/s00726-023-03334-w PMC10689525

[50] Troha K, Ayres JS (2020) Metabolic Adaptations to Infections at the Organismal Level. Trends Immunol 41(2):113–125 31959515 10.1016/j.it.2019.12.001 PMC7409656

[51] Cuesta S et al (2022) Gut colonization by Proteobacteria alters host metabolism and modulates cocaine neurobehavioral responses. Cell Host Microbe 30(11):1615-1629.e5 36323315 10.1016/j.chom.2022.09.014 PMC9669251

[52] Om H, Chand U, Kushawaha PK (2023) Human anaerobic microbiome: a promising and innovative tool in cancer prevention and treatment by targeting pyruvate metabolism. Cancer Immunol Immunother 72(12):3919–3930 37882845 10.1007/s00262-023-03551-y PMC10992366

