# Supplementary figures and images for: Commensal Peptoniphilus harei induce activation of monocytes via TLR2/CD14 signalling in whole blood
Source: Med Microbiol Immunol. 2025 Nov 27;214(1):52. doi: 10.1007/s00430-025-00859-7 (PMC12660362; doi:10.1007/s00430-025-00859-7)

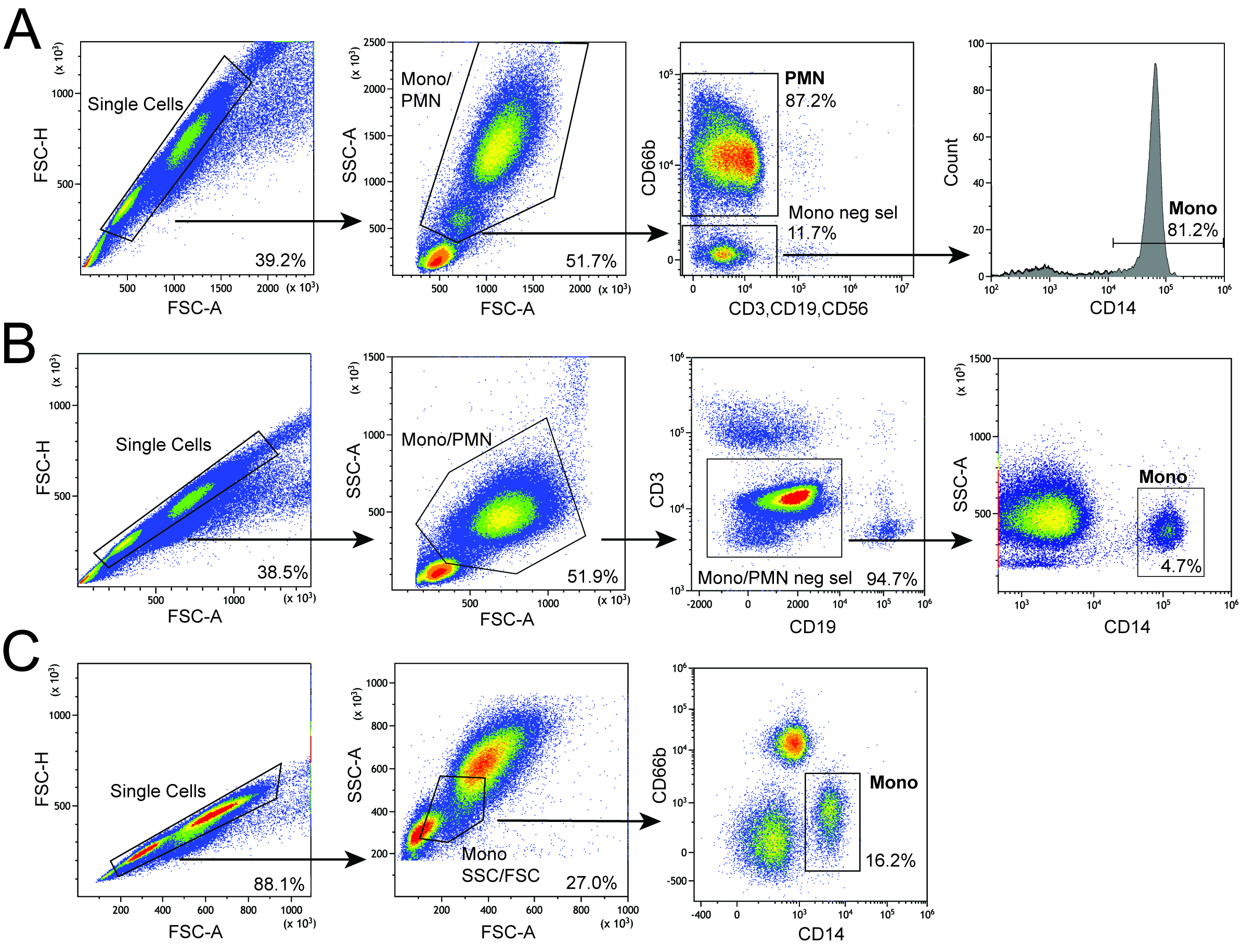

Supplement: Supplementary file 1 — Supplementary Material 1 [file 430_2025_859_MOESM1_ESM.tiff]

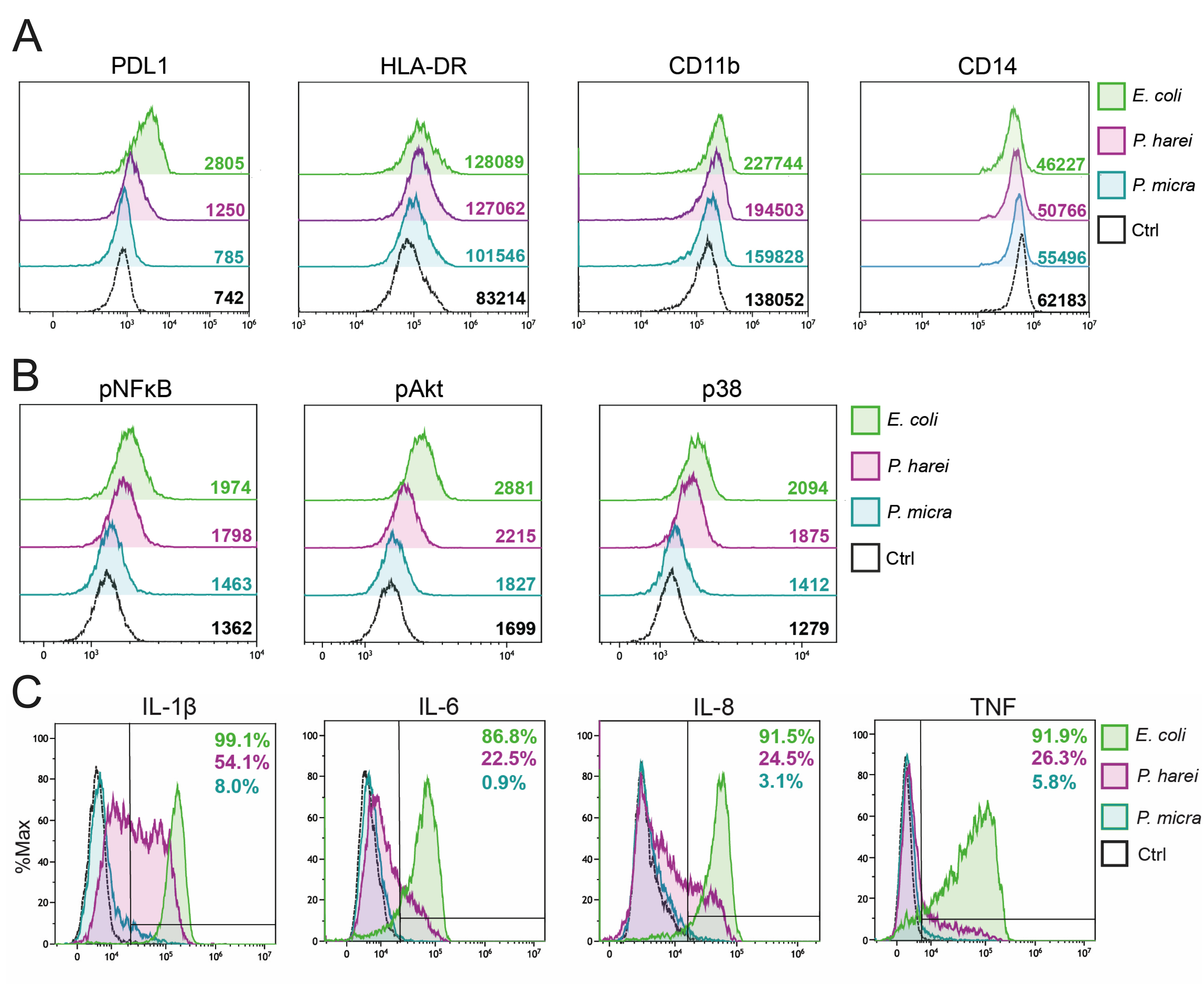

Supplement: Supplementary file 2 — Supplementary Material 2 [file 430_2025_859_MOESM2_ESM.tiff]

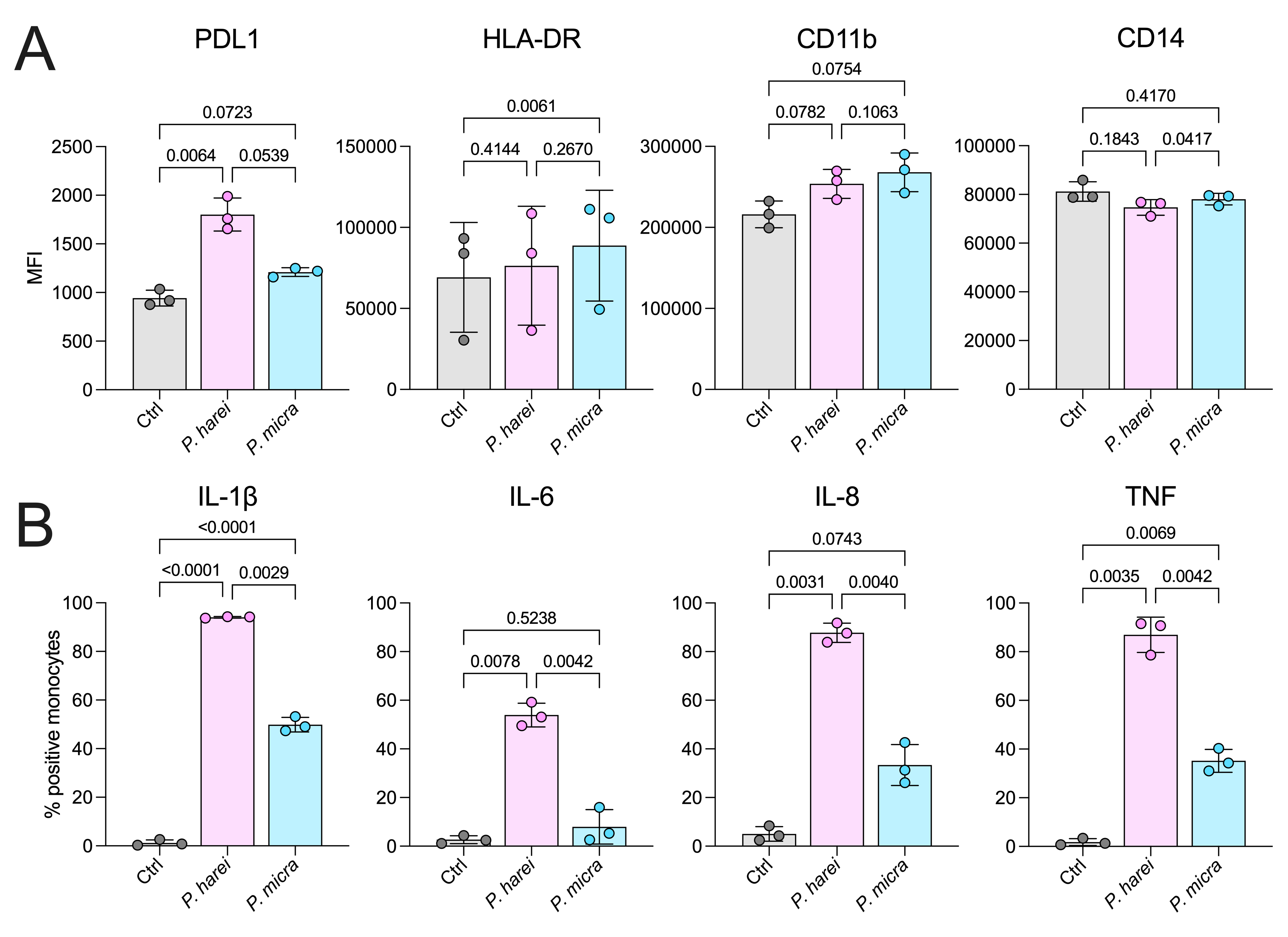

Supplement: Supplementary file 3 — Supplementary Material 3 [file 430_2025_859_MOESM3_ESM.tiff]

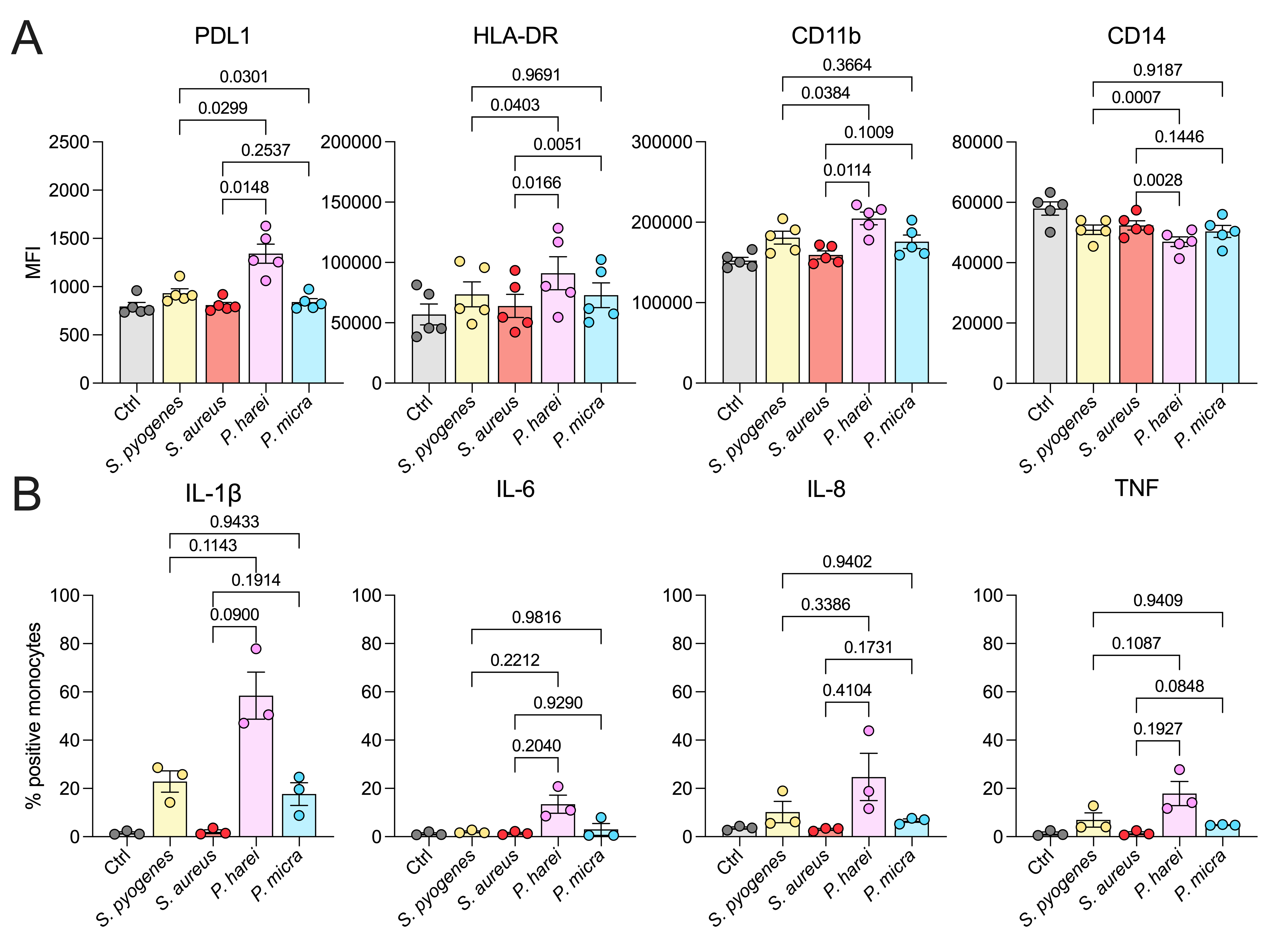

Supplement: Supplementary file 4 — Supplementary Material 4 [file 430_2025_859_MOESM4_ESM.tiff]

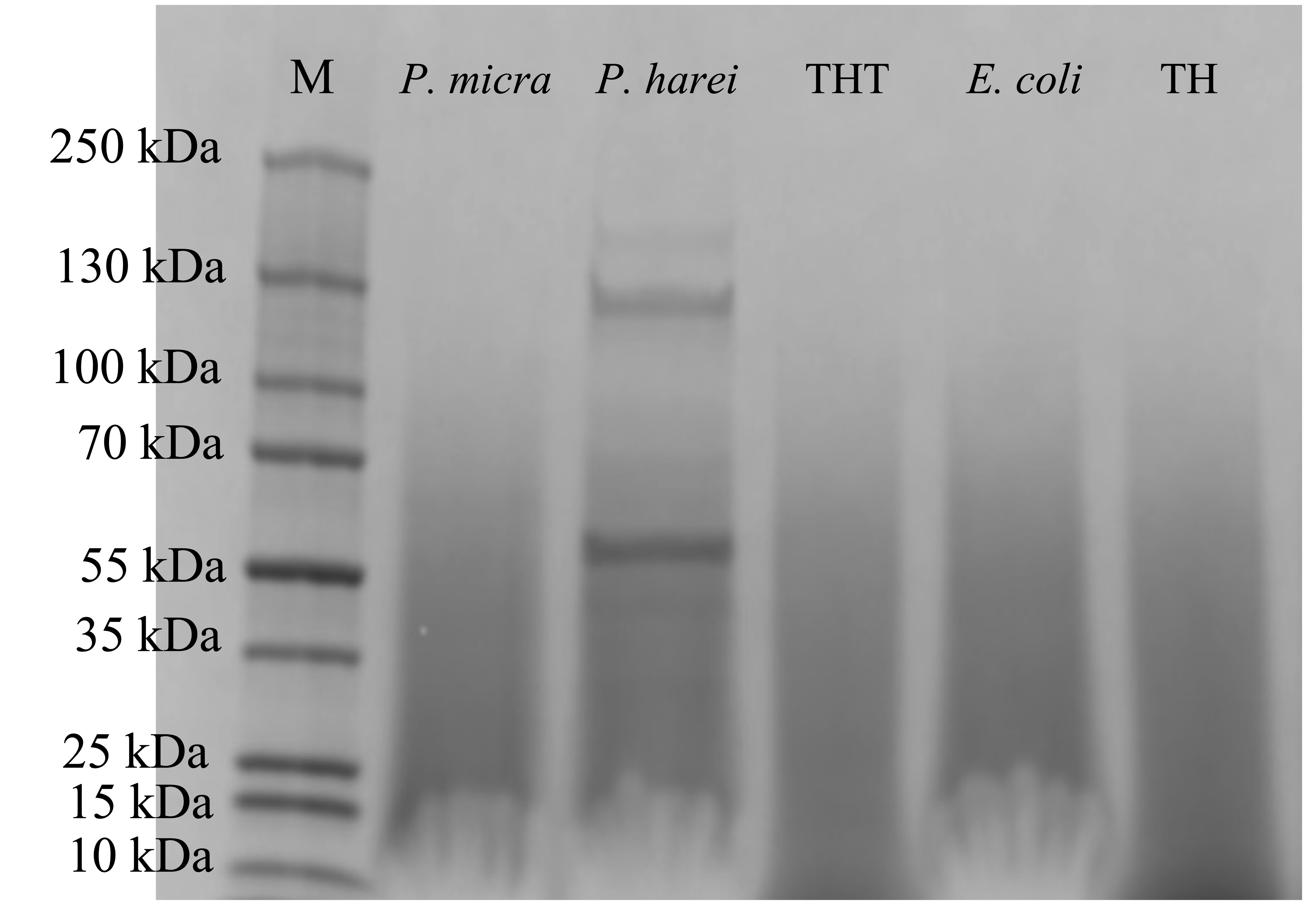

Supplement: Supplementary file 5 — Supplementary Material 5 [file 430_2025_859_MOESM5_ESM.tiff]
